# Surviving With Mycosis Fungoides for Twenty Years: An Autobiographical Case Report

**DOI:** 10.7759/cureus.20699

**Published:** 2021-12-25

**Authors:** Dillip Kumar Parida, Sandip Kumar Barik

**Affiliations:** 1 Radiation Oncology, All India Institute of Medical Sciences, Bhubaneswar, Bhubaneswar, IND; 2 Radiation Oncology, All India Institute of Medical Sciences, Bhubaneshwar, Bhubaneswar, IND

**Keywords:** radiotherapy, skin cancers, hdr electron therapy, mycosis fungoides, total skin body electron therapy, cutaneous lymphoma

## Abstract

Mycosis fungoides (MF) is a low-grade chronic lymphoid proliferative disorder of T-lymphocytes arising out of the skin, having an indolent course caused by abnormal proliferation of CD4+ T-cells. Here we present a case of a 37-year-old male who was diagnosed with mycosis fungoides in 2001 and treated with Total Skin Electron Beam Therapy (TSEBT). The purpose of this autobiographical case report is to give an insight into the eventful journey of the patient living with the disease for the last 20 years. His journey will serve the purpose of both patients and physicians and will add to the literature on the subject.

## Introduction

Mycosis fungoides (MF) is a low-grade chronic lymphoid proliferative disorder of T-lymphocytes arising out of the skin, having an indolent course caused by abnormal proliferation of CD4+ T-cells [1]. Histopathologically, MF is characterized by an epidermotropic infiltrate of T-lymphocytes that display, in most cases, a helper phenotype [2]. The classical MF progresses through five phases: premycotic, patch, plaque, tumor, and erythroderma [3]. Skin-directed treatments are preferred in patients with early-stage disease in the form of topical corticosteroids, phototherapy, superficial radiotherapy, and nitrogen mustard preparations. Radiation therapy remains the treatment of choice as the cutaneous lesions are radiosensitive [4,5]. Total Skin Electron Beam Therapy (TSEBT) is usually delivered by 4-6 MeV electrons to a total dose of 36Gy.

Here we present a case of a 37-year-old male who was diagnosed with mycosis fungoides in 2001 treated with TSEBT. Currently, the patient is in normal health, and this case report describes the eventful journey of the patient living with the disease for the last 20 years.

## Case presentation

**Figure 1:**
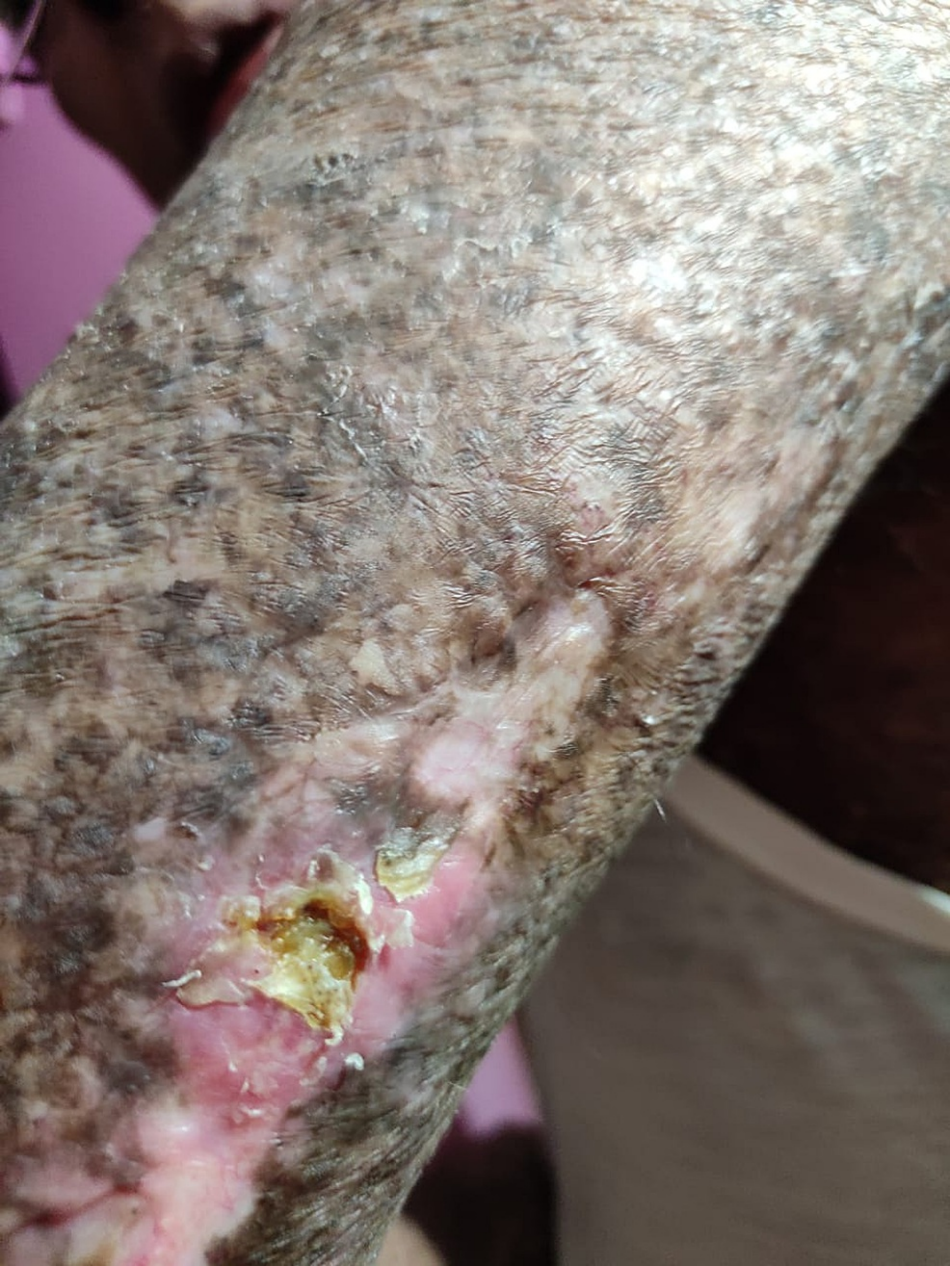
Shows ulceration present over the right forearm post-treatment with TSEBT. TSEBT: Total Skin Electron Beam Therapy

The patient was diagnosed with mycosis fungoides in 2001. On presentation, the patients had nodular lesions over the waistline and papular patches all over the body. Biopsy taken from the lesion suggested MF. The patient was treated with TSEBT on High Dose Rate (HDR) mode with modified fractionation scheduled in 2001. A total dose of 36Gy over 9 weeks was delivered with a daily fractionation of 120cGy. Post-treatment, the patient had a clinically controlled disease with few side effects. In 2011 he developed contracture of the right elbow joint, a nonhealing ulcer, and a plaque over the contracture in 2012. The ulcer was of a size of 4x4 cm over the radial aspect of the right forearm. Biopsy from the margin of the ulcer showed features of squamous cell carcinoma (SCC) (Figure 1). While biopsy from the plaque showed orthokeratosis, irregular acanthosis in the epidermis and dermis shows few melanophages. The newly developed SCC was treated by local excision and repair of the triceps tendon. In 2015 patient developed a few areas of hypopigmentation over the body with a few areas of hypopigmentation in the back of arms. In 2019 again, a nonhealing ulcer developed over the right arm, which was treated with antibiotics and healed subsequently. Biopsy from the lesion was negative for malignancy. Currently, the patient has a clinically controlled disease, with a survival of 20 years, although with treatment-related morbidity.

## Discussion

Mycosis fungoides (MF) is the most common type of cutaneous lymphoma, representing almost 50% of all lymphomas arising primarily in the skin [6]. The disease is more common in adults and elderly patients with a male to female ratio of 2:1 [6,7 ], but it also can be observed in children and adolescents [8]. MF is divided into three clinical phases: patch, plaque, and tumor stage, and the clinical course is usually protracted over years or decades [9]. MF typically starts as flat and scaly pink or red patches on the hands, chest, abdomen, upper thighs, or buttocks. Early-stage patients may progress slowly over many years and may progress to an advanced stage with tumors, erythroderma, lymph node, or visceral involvement that may occur with a poor prognosis [10]. The frequency of occurrence of cutaneous lymphomas was found to be 0.7 per 100 biopsy specimens contributing about 73% of all lymphomas [11]. Histopathologically, MF is characterized by an epidermotropic proliferation of small- to medium-sized pleomorphic lymphocytes forming intraepidermal collections called Pautrier's microabscesses and is considered the histopathological hallmark of the disease [12]. The principal prognostic factors are the extent and phase of the disease, lymphadenopathy and visceral involvement, and the presence of lymphoma cells in the peripheral circulation [13].

Radiotherapy has been used for many years and is a treatment of choice for patients with MF. Radiotherapy is an effective treatment for early-stage disease and can palliate symptoms when the disease is advanced [14]. The complete remission rates with this therapy may be as high as 90-100% in localized lesions. Cotter et al. demonstrated 100% remission with a radiation dose above 30 Gy [15]. Wilson et al. have reported a remission rate of 97% with external beam radiotherapy [16]. A total dose of 36-40 Gy of radiation over 10-11 weeks, with 2-5 days a week treatment, is commonly used. TSEBT is technically challenging and should be done in equipped centers with an experienced team. Diagnosis of MF is complex, and considering time consumption for TSEBT, and other technical requirements, many centers in the world and India are not using this treatment. Modern TSEBT is usually accomplished with 4-6 MeV electrons generated by a medical linear accelerator directed at a patient behind a polycarbonate screen, about 10 feet from the linear accelerator head. Still, areas such as the soles of the feet, perineum, and scalp remain underdosed and can be given a boost dose by electrons separately. In a study by Parida and Rath in 2013, TSEBT was carried out using 4 MeV High Dose Rate (HDR) electrons [17]. The HDR mode facility delivers a high dose rate of about 30 Gy/min, substantially decreasing the treatment time. In HDR, patient positioning should be strictly maintained throughout the treatment to prevent any target miss or get a high dose to organ at risk. A total dose of 36 Gy was delivered over 9-14 weeks with daily fractionation of 120 cGy along with a booster dose of 10 Gy to the scalp, perineum, and sole.

Due to acute and late side effects, TSEBT, a promising tool, could not become a popular choice [18]. Due to the long treatment duration and the technical challenges in implementing the TSEBT is not widely practiced in India. Acute side effects are erythema, edema, hyperpigmentation, and fatigue. Additional acute side effects commonly include xerosis, dry desquamation, extremity edema, blister/bullae formation, alopecia, nail changes, dryness of nasal mucosa, and hypohidrosis secondary to damaged sweat glands may occur. Late/chronic side effects include cataract formation, xerosis, alopecia, nail changes, telangiectasia, and secondary skin cancers. Like the above findings, our patient discussed in the case reports also had erythema and hyperpigmentation. Later, he developed SCC from the right forearm, which was treated with electrons previously. During treatment of TSEBT, sensitive parts like nails, eyes, lips, hair, and testis can be shielded as loss of these skin appendages invariably occurs by the end of treatment. In our therapy with TSEBT, the patient's eye and nails were shielded from day 1 of treatment. Chronic cutaneous damage from TSEBT irradiation is unusual at doses of <10 Gy and is acceptably mild through 25 Gy. TSEBT is very effective, particularly for early-stage disease. TSEBT yields a Complete Response (CR)rate of >90% with a 15-year relapse-free survival of 40% in patients with T1 disease [19], and in the T2 condition, TSEBT is similarly effective, with a CR rate of 76% to 90% and a 50% relapse-free survival rate at 5 years and 10% at 10 years. In T3 disease, TSEBT is less effective, with a CR rate of 44% to 54% of patients [19 ]. Our patient had a clinically controlled disease and has a survival of 20 years.

## Conclusions

Living and fighting MF successfully is equally challenging for the patient and the physician. Both have different problems that must be addressed to benefit many patients suffering from MF. Post-treatment with TSEBT, the patient has to live with many serious side effects, including skin cancers. This case report will serve the purpose of both patient and physician as it depicts the patient's journey for 20 years and will add to the literature on the subject.
